# How do patient reported outcome measures (PROMs) support clinician-patient communication and patient care? A realist synthesis

**DOI:** 10.1186/s41687-018-0061-6

**Published:** 2018-09-15

**Authors:** Joanne Greenhalgh, Kate Gooding, Elizabeth Gibbons, Sonia Dalkin, Judy Wright, Jose Valderas, Nick Black

**Affiliations:** 1 School of Sociology and Social Policy, University of Leeds, Woodhouse Lane, Leeds, LS2 9JT England; 2 Present address: Liverpool School of Tropical Medicine, Pembroke Place, Liverpool, L3 5QA UK; 3 Nuffield Department of Population Health, University of Oxford, Richard Doll Building, Old Road Campus, Oxford, OX3 7LF UK; 4 Present address: Department of Social Work, Education & Community Wellbeing, Northumbria University, H005, Coach Lane Campus East, Newcastle upon Tyne, NE7 7XA England; 5 Leeds Institute of Health Sciences, University of Leeds, Worsley Building, Clarendon Way, Leeds, LS2 9NL England; 6 Health Services and Policy Research, Exeter Medical School, University of Exeter, St Luke’s Campus, Heavitree Road, Exeter, EX1 2LU England; 7 Health Services Research, London School of Hygiene and Tropical Medicine, 15-17 Tavistock Place, London, WC1H 9SH England

**Keywords:** Patient reported outcome measures, Realist synthesis, Clinician-patient communication, Feedback

## Abstract

**Background:**

In this paper, we report the findings of a realist synthesis that aimed to understand how and in what circumstances patient reported outcome measures (PROMs) support patient-clinician communication and subsequent care processes and outcomes in clinical care. We tested two overarching programme theories: (1) PROMs completion prompts a process of self-reflection and supports patients to raise issues with clinicians and (2) PROMs scores raise clinicians’ awareness of patients’ problems and prompts discussion and action. We examined how the structure of the PROM and care context shaped the ways in which PROMs support clinician-patient communication and subsequent care processes.

**Results:**

PROMs completion prompts patients to reflect on their health and gives them permission to raise issues with clinicians. However, clinicians found standardised PROMs completion during patient assessments sometimes constrained rather than supported communication. In response, clinicians adapted their use of PROMs to render them compatible with the ongoing management of patient relationships. Individualised PROMs supported dialogue by enabling the patient to tell their story. In oncology, PROMs completion outside of the consultation enabled clinicians to identify problematic symptoms when the PROM acted as a substitute rather than addition to the clinical encounter and when the PROM focused on symptoms and side effects, rather than health related quality of life (HRQoL). Patients did not always feel it was appropriate to discuss emotional, functional or HRQoL issues with doctors and doctors did not perceive this was within their remit.

**Conclusions:**

This paper makes two important contributions to the literature. First, our findings show that PROMs completion is not a neutral act of information retrieval but can change how patients think about their condition. Second, our findings reveal that the ways in which clinicians use PROMs is shaped by their relationships with patients and professional roles and boundaries. Future research should examine how PROMs completion and feedback shapes and is influenced by the process of building relationships with patients, rather than just their impact on information exchange and decision making.

**Electronic supplementary material:**

The online version of this article (10.1186/s41687-018-0061-6) contains supplementary material, which is available to authorized users.

## Introduction

The clinician-patient relationship has been an enduring focus of research across many disciplines and has received considerable attention from policy makers internationally. Efforts to increase patient involvement in decision making about their care [1, 2] are expected to improve patient well-being and health outcomes [3]. Changing the ways in which clinicians and patients communicate with each other, in particular, the practice of patient centred communication, is cast as one of the mechanisms through which these outcomes will be realised [4, 5]. The completion of patient reported outcome measures (PROMs) by patients and the feedback of these data to clinicians is one intervention that has been argued to support communication between clinicians and patients and, in turn, improve care processes and outcomes [6, 7].

There are many quantitative systematic reviews of PROMs feedback in the care of individual patients but they have struggled to draw definitive conclusions about its impact [8–12]. Most reviews limited their inclusion criteria to randomised controlled trials (RCTs) to examine whether (rather than how or why) PROMs feedback ‘works’. One pattern evident in quantitative reviews is that PROMs feedback has a greater impact on clinician-patient communication, the provision of advice or counselling and the detection of problems than on patient management and subsequent patient outcomes. However, why and how this pattern of impact occurs has rarely been explored [13]. More recently, reviews have included qualitative studies of clinician and patient experiences of using PROMs to synthesise evidence on their implementation and use [14–16]. While these reviews provide a useful summary of barriers and facilitators, they do not explain why barriers in one context may be facilitators in another [15] nor the mechanisms through which these barriers or facilitators work. An alternative review methodology is required that can address this complexity.

In this paper, we report the findings of a realist synthesis that aimed to understand how and in what circumstances PROMs support patient-clinician communication and subsequent care processes and outcomes in clinical care. First, we describe realist synthesis and the methodology of the review. Second, we outline the programme theories which constitute the anticipated mechanisms through which PROMs may (or may not) support clinician-patient communication and subsequent care processes. Thirdly, we present the findings of our evidence synthesis. Finally, we discuss our findings in the context of broader debates about how respondents make sense of survey items and clinician-patient communication and consider the implications of our findings for future research and clinical practice.

## Methodology

Realist synthesis is a review methodology based on the premise that interventions constitute ideas and assumptions, (programme theories), about how and why they are supposed to work [17]. Interventions offer (or remove) resources and the ways in which participants respond to these resources (mechanisms) determine the outcomes of the intervention. These responses are shaped by the design of the intervention itself and the circumstances into which the programme is implemented (context). Our realist synthesis aimed to identify, test and refine programme theories to build explanations about how context shapes the mechanisms through which PROMs use may support clinician-patient communication, subsequent care processes and why. Our protocol was published [18] and was registered with PROSPERO (registration number 42013005938). We followed the RAMESES I guidelines to report the synthesis [19]. A full report of the synthesis was published by the funder in the NIHR journals library [20]. This paper builds on this report in three important ways. First, it offers an extended analysis of existing literature by a closer interrogation of context and outcome patterns in an existing systematic review [8]. Second, it provides an updated review of recent literature by conducting further searches to capture studies published since 2016; nine additional papers were included in the synthesis [21–29]. Third, it advances the interpretation of our findings by drawing on theory from the philosophy of measurement [30] and current debates in clinician-patient communication [31].

**Fig. 1:**
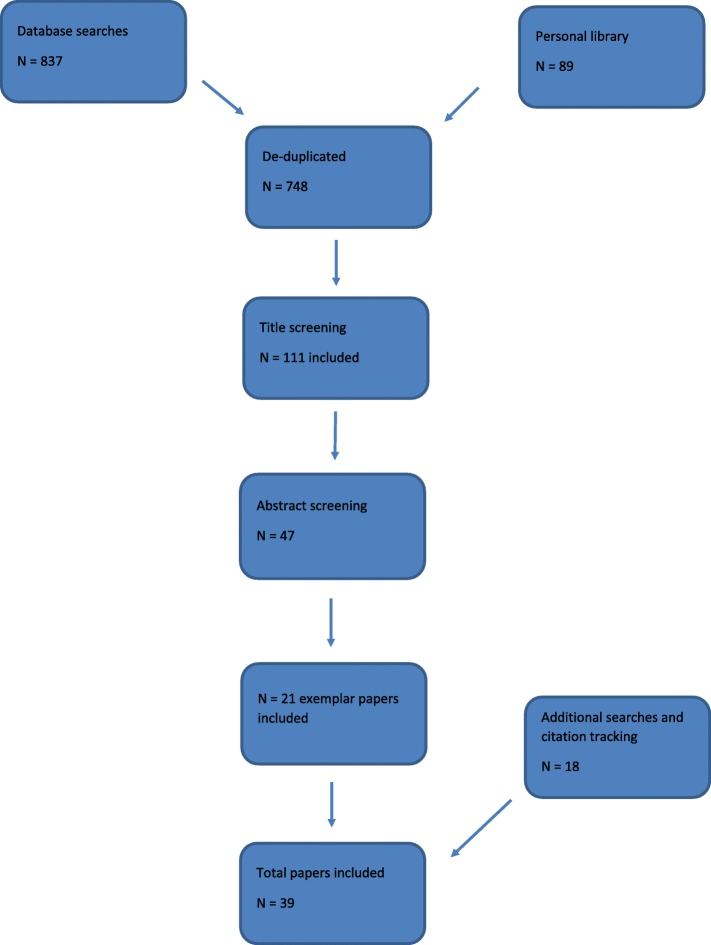
Selection of papers that contributed to the development of the initial programme theories

Realist synthesis is iterative but for simplicity we describe it here as having two main phases: (1) theory identification and (2) theory testing and refining. The first phase of our synthesis sought to identify the programme theories underlying the use of PROMs in clinical practice. We identified these theories through an analysis of policy documents, commentaries, reviews, comments, letters, and editorials. Papers were found drawing on references used to write the research funding proposal (‘personal library’), searches of electronic databases in October 2014 (search strategy in Additional file 1) and citation tracking of these papers. In total, 39 papers contributed to the development of the initial programme theories (Fig. 1). Other screened papers also contributed to our thinking; however, the 39 ‘included’ papers were deemed to provide the clearest exemplars of programme theory in the final synthesis. We also held a two-hour workshop with clinicians, managers, policy-makers and patients to verify and expand the theories.

The second phase of our synthesis involved testing and refining these programme theories through an examination of empirical studies to understand how, why and in what circumstances the purported benefits of PROMs are realised in practice. We identified this empirical evidence as follows. We used forward citation searches of six key [10, 11, 13, 15, 32, 33]. These included four systematic reviews [10, 11, 13, 15] that were chosen because they represented the use of PROMs across different care settings and incorporated quantitative, qualitative and theory driven reviews. These key papers also included a qualitative study chosen because it explicitly explored the use of PROMs within clinician-patient interactions [32] and a study of the use of needs assessments in health visiting [33], chosen because it was an intervention which shared similar programme theories but drew on sociological theories to interpret the findings. We also searched the reference lists of five systematic reviews [8, 11, 13, 15, 34] which represented a range of clinical contexts and review methodologies and one of the above key papers [32]. These searches were undertaken using Web of Science Core Collection Citation Indexes (Thomson Reuters) in May 2015. As the synthesis progressed, we conducted supplementary searches including key author searches and additional citation tracking of key papers and systematic reviews to identify related studies [35, 36]. In February 2018, we updated the forward citation tracking of the six key papers [10, 11, 13, 15, 32, 33] using both Scopus and Google Scholar citation tools to capture studies published since the original searches were carried out. We also conducted a forward citation search for a systematic review the played a key role in testing theory 2 [8].

Study selection, data extraction, quality assessment, synthesis and additional literature searching occurred simultaneously. Study selection was undertaken by JG and discussed and agreed with KG and EG. Studies were selected on the basis of their contribution to theory testing using a set of inclusion and exclusion criteria as a guide (Additional file 2). In some instances, the whole study contributed to theory testing and in others, only a fragment or fragments of the study were relevant to the theory. Each fragment of evidence was appraised, as it was extracted, for its relevance to theory testing and the rigour with which it has been produced [37]. The rigour of randomised controlled trials were assessed using the Cochrane risk of bias tool [38]. Formal checklists were not used to appraise the qualitative studies; as others have noted, qualitative research methods vary widely and rigour in qualitative studies is often not reducible to technical fixes and checklists [39–41]. Therefore, quality appraisal represented a judgement based on the particular methods used and related specifically to the validity of the causal claims made in these subset of findings, rather than the study as a whole. Trust in these causal claims was also enhanced by the accumulation of evidence from a number of different studies which provide further lateral support for the theory being tested. Papers were summarised using a data extraction template to facilitate cross and within analysis of the papers. The ongoing synthesis was discussed at regular meetings with wider project group (JMV, NB and SD).

**Fig. 2:**
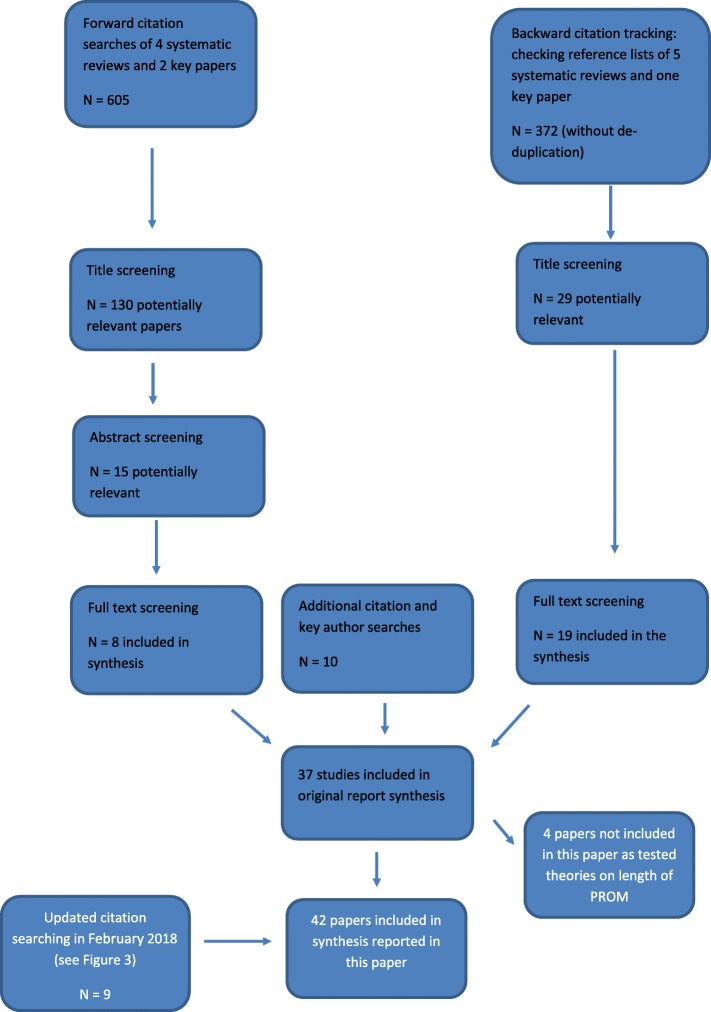
Process of paper review and selection for synthesis reported in this paper

**Fig. 3:**
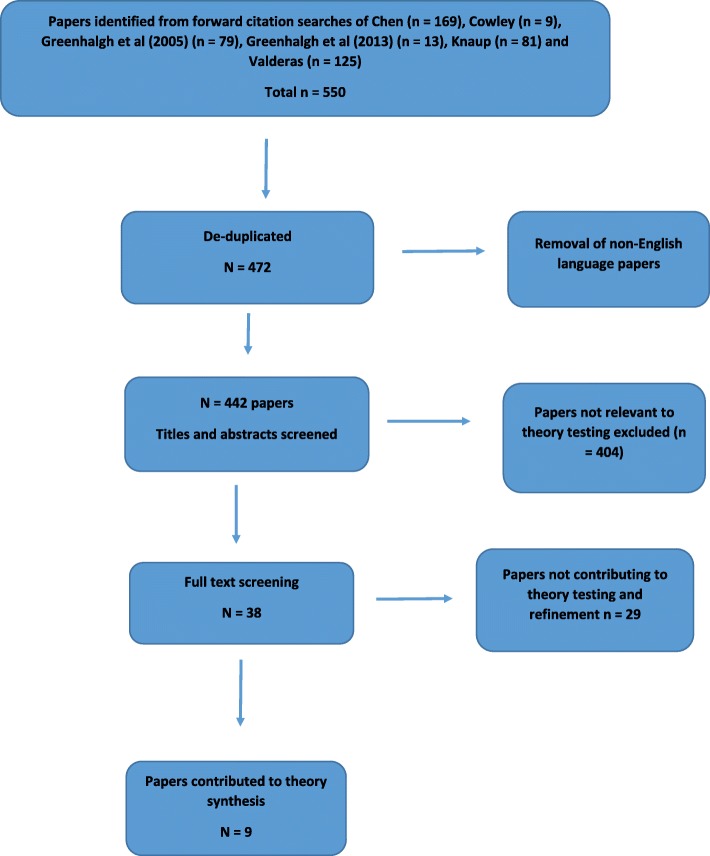
Selection of papers from citation searches conducted in February 2018

In total, 46 papers were included in the evidence synthesis of which 42 are drawn on for this paper (Table 1); the remaining four tested theories about patients’ views on the length of PROMs, which is not discussed in this paper. Figure 2 shows how the papers were selected for our original report (*n* = 37) and Fig. 3 shows how the papers were selected from our February 2018 forward citations searches (*n* = 9). Details of how we synthesised the evidence for each theory are provided in the ‘Findings: Theory Testing and Refining’ section.

## Findings: Theory identification

### ‘Positive’ programme theories

**Fig. 4:**
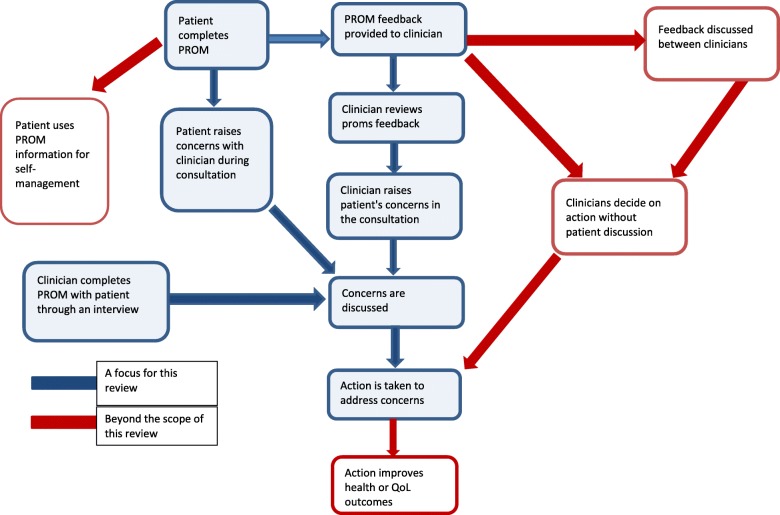
PROMs feedback in the care of individual patients: Implementation chain at start of synthesis

The review of programme theories indicated several hypothesised roles for PROMs in the care of individual patients that have evolved and changed over time [6, 42, 43]. These include identifying patients with anxiety or depression, assessing patient needs, monitoring the outcomes and side effects of treatment, informing goal setting, supporting shared decision making and enabling patients to self-manage long term conditions [44]. Drawing on previous work [13, 45], we developed an initial diagram of the PROMs feedback ‘implementation chain’ to illustrate the pathways through which proximal, intermediate and distal outcomes are thought to be achieved (Fig. 4). We decided that self-tracking using PROMs to support patient self-management [46] and use of PROMs by clinicians without discussion with patients were beyond the scope of the review and concentrated on how PROMs supported interactions between patients and clinicians. Previous systematic reviews of PROMs feedback suggested there is a ‘blockage’ between communication and action along these pathways [8, 47]. Therefore we focused on understanding how PROMs feedback supports clinician-patient communication and subsequent care processes; we wanted to understand what happens ‘inside the arrows’ shown in Fig. 4. We identified two dominant, overarching programme theories about the mechanisms through which PROMs might support clinician-patient communication, though each theory also encapsulated a number of ‘sub’ theories:

#### Theory 1: PROMs completion supports patients to raise issues with clinicians

We identified several mechanisms through which this may occur. PROMs completion may prompt patients to engage in a process of self-reflection and help them to identify what is important to them [45, 48, 49]. It may also empower patients or give them ‘permission’ to raise issues with clinicians [45, 48].

#### Theory 2: PROMs scores raise clinicians’ awareness of patients’ problems

PROMs may offer a systematic and comprehensive assessment of patients’ perceptions of their symptoms, functioning or HRQoL. PROM scores alert clinicians to issues that, it is assumed, they were previously unaware of [50–52]. This is expected to prompt clinicians to explore these issues with patients; implicit here is that clinicians value and discuss patient’s experience as well as biomedical information [53, 54].

These theories are not necessarily mutually exclusive but represent different stages of the implementation chain (Fig. 2) and constitute different understandings of the function of PROMs. Theory 1 emphasises the *process* of PROMs completion whereas in theory 2, the PROM *score* is assumed to act like a test result, similar to biomedical indicators. In turn, these are expected to support care processes.

### ‘Counter’ programme theories

We identified a number of ‘counter’ or ‘opposing’ programme theories that challenged the assumptions underlying the theories discussed above. First, some queried whether the content and structure of standardised PROMs adequately capture and reflect patients’ views [49, 55, 56]. Second, the assumption that clinicians do not effectively elicit information from patients or insufficiently engage with their emotional cues has been questioned [57–59]. Third, it has been argued that PROMs may not provide clinicians with any new information about patients over and above talking to them [59, 60]. For example, Salander [57] observes there is a fundamental difference between (for example) screening for breast cancer and screening for psychological distress. The presence of a tumour is a ‘biomedical fact’ that was hitherto unknown to the patient whereas the insight revealed from a distress questionnaire is dependent on what a patient chooses to disclose; disclosure that, it is argued, could also occur through open dialogue. Finally, PROMs may offer an inferior substitute for meaningful nuanced communication and their structured nature may divert attention away from patients’ concerns and reinforce the clinicians’ agenda [59, 61].

### Context

The programme theories above largely focus on mechanisms and some draw on models that elucidate the pathways through which communication is anticipated to influence patient care and outcomes [62, 63]. However, such models often reduce complex social processes to variables, assume a linear, universal relationship between them and do not consider how context may shape these pathways [31, 61, 64]. We wanted to understand what contextual conditions are hypothesised to shape the ways in which PROMs may support clinician-patient communication to inform the process of theory testing within our evidence synthesis.

First, the content and structure of the PROM and the ways it is administered and fed back is likely to shape its impact on clinician-patient communication and subsequent care processes. Although many PROMs blur the two, clinicians may respond differently to information on patients’ symptoms compared to data on HRQoL [59]. It has been argued that individualised PROMs may be more appropriate than standardised PROMs for use in routine clinical practice, as they allow patients to nominate domains of most relevance and indicate the relative importance of each domain [119, 120]. The process of completing individualised instruments such as the Schedule for the Evaluation of Individual Quality of Life (SEIQoL) is envisaged to provide the ‘therapeutic foundation’ for goal setting and developing the clinician-patient relationship [121]. However, others note the requirement to distil complex and dynamic experiences into a score renders such measures as reductionist as standardised measures [49].

Second is the care setting or context; this encompasses a number of interrelated sets practices, norms and relationships that may shape the how PROMs are used and how participants respond [65]. Salmon and Young [31] highlight differences between care settings in the extent to which clinicians are expected to emotionally engage with patients. For example, in a mental health context, there is a presupposition that clinicians engage with patients emotionally as part of the care and treatment process whereas in an oncology context, patients expect their oncologist to cure or manage their tumour in order to preserve life. However, others consider that recognising and explicitly responding to patient’s emotional concerns is central to the care of cancer patients [62]. Related to this, Lafata et al. [64] observe that the purpose of care and thus the nature of clinician-patient communication changes over time throughout a patient’s journey, for example, in cancer care, from screening, treatment and advanced cancer care. Thus, PROMs are likely to play a different role in supporting clinician-patient communication during initial assessments compared to during active treatment or during end of life care. Finally, this is also framed by differences in care delivery across settings; for example, in specialist mental health care, patients may see the same therapist over time whereas in cancer care, they may see different oncologists and different healthcare professionals. This also influences the nature of the relationships and the relationship building process between patients and clinicians across settings.

## Findings: Theory testing and refining

### Synthesis: Testing the theories

In the second phase of our synthesis, we sought to explore whether, how and why the programme theories and counter theories are realised in practice by reviewing empirical evidence and refine our theories in light of that evidence. To test and refine theory 1, we compared the findings of studies examining clinicians’ and patients’ experiences of using either or both standardised and individualised PROMs across three different settings (primary care, specialist mental health care and cancer care), which we theorised represented a range of care contexts and in particular, different configurations of care delivery and clinician-patient relationships. Much of the evidence we reviewed to test this theory involved the use of PROMs within the care encounter or where clinicians were responsible for approaching patients to request they complete a PROM. To test theory 2, we focused on oncology as there is a high volume of literature [8, 16, 47, 66] and it has been the focus of debate regarding the role of explicit emotional communication in the care of patients [31]. We examined how PROMs influence communication and subsequent care, drawing on systematic reviews and RCTs, qualitative studies and studies of interactions in oncology consultations. Most of the RCTs involved PROMs completion outside the consultation. Table 1 sets out how each included study contributed to the process of theory testing and refinement.

**Table 1:** Studies used to test theories 1 and 2

Authors	Description of PROM use	Aims and study design	Setting			Function of PROM		Type of PROM		Key findings that contributed to theory testing
			Primary care	Specialist mental health	Oncology or Palliative care	Screening/ Assessment	Monitoring	Standard	Individual	
Testing Theory 1										
Studies exploring patients’ experiences of completing a PROM outside of routine use in clinical settings										
Neudert et al. [80]	Completion of Sickness Impact Profile, SF-36 and SEIQOL-DW by 42 patients with amyotrophic lateral sclerosis in Germany	All patients completed the Sickness Impact Profile; patients randomly allocated to complete SF-36 or SEIQOL-DW. Questionnaire to assess patient’s perceptions of validity and distress caused by completion						✓	✓	• Patients’ ratings for the perceived validity of the instrument in measuring their quality of life were significantly higher for the SEIQOL-DW than the SIP or SF-36. • The SEIQoL-DW had the lowest rating for emotional distress, followed by the SF-36 and then the SIP, with the difference between the SEIQoL-DW and the SIP being statistically significant.
Mallinson [85]	Completion of SF-36 by 56 people over 65 referred for physiotherapy in UK	Qualitative analysis of standardised interviewer administration of the SF-36						✓		• Technical issues included double barrelled questions, use of unfamiliar or vague terms. • Conceptual issues included: items premised on implicit assumptions about what is ‘normal’ that was not shared by respondents.
Westerman et al. [81]	Completion of SEIQOL-DW by 31 patients with small cell lung cancer in the Netherlands	Qualitative analysis of cue elicitation process during interviewer administration of SEIQOL-DW.			✓				✓	• Cues are co-constructed by interviewers and patients. • Reducing patient narratives into five cues can result in loss of meaning
Farquhar et al. [82]	Completion of SEIQOL-DW by 13 patients with COPD in the UK	Qualitative analysis of three step process of interviewer administration of SEIQOL-DW.							✓	• Cue selection can distort patient experiences. • Patients used different frames of reference to rate severity of cues and misunderstood instructions.
Studies exploring the use of PROMs to detect and manage patients with depression in primary care settings										
Dowrick et al. [65]	Use of standardised PROMs to detect patients with depression in UK	Qualitative interviews with 24 patients and 34 GPs to explore their experiences of PROM use	✓			✓		✓		• Some patients felt PROMs completion helped them to articulate their feelings and communicate these to GP • Completing a PROM made some patients feel GP was taking them seriously. • PROMs completion helped some patients reflect and increase their understanding of their condition. • Some patients found PROMs didn’t reflect complexity of their condition.
Leydon et al. [75]	Use of standardised PROMs to detect patients for depression in UK	Qualitative interviews with 24 patients and 34 GPs to explore their experiences of PROM use	✓			✓		✓		• GPs felt developing a relationship with patients was important to detect and manage depression. • GPs perceived PROMs as mechanistic and felt they trivialised patients’ emotions. • GPs felt uncomfortable asking patients to complete a PROM and found it difficult to integrate them into flow of consultation.
Mitchell et al. [83]	Use of standardised PROMs to detect patients for depression in UK	Four qualitative focus groups to explore primary care practitioners experiences of PROMs use	✓			✓		✓		• GPs felt PROMs were intrusive and duplicated what they already knew about patients, so did not influence management. • GPs adapted the way they administered the PROM to fit into the flow of clinical work in ways that sometimes compromised the validity of the PROM.
Pettersson et al. [76]	Use of standardised PROMs to detect and manage patients for depression in Sweden	Qualitative interviews with 27 GPs to explore their experiences of PROM use	✓			✓		✓		• GPs perceived PROMs did not add to what they already gathered from patients by verbal interaction. • GPs suggested PROMs distracted them from noticing patient’s non-verbal communication. • Most GPs felt PROMs constrained the narrative of the patient, interfered with their ability to listen to the patient.
Studies exploring PROM use in specialist mental health care services										
Hall et al. [70]	Pilot study of session by session outcome monitoring using the Strengths and Difficulties questionnaire in Children and Young People’s Improving Access to Psychological Therapies programme in the UK	Qualitative interviews with 10 clinicians, 8 administrative staff and 15 families to explore their views and experiences of PROMs use		✓			✓	✓		• Clinicians felt completing PROMs during the session got in the way of the therapeutic process while referring to results created awkward silences and distracted patients. • Families felt completing the PROM was helpful in its own right as it provided an opportunity to reflect and assess their condition. • Patients felt PROMs completion helped them to understand their progress if clinicians discussed findings with them.
Wolpert et al. [74]	Child mental health services prior to implementation of a symptom checklist; childrens’ diabetes services currently using the PedsQL in the UK	Focus groups with 5 children accessing mental health services; 4 interviews with young people and their mothers accessing diabetes services; 3 interviews with mothers whose children accessed diabetes services to explore views of PROMs		✓			✓	✓		• Children held mixed views about value of PROMs and questioned whether they could capture the dynamic nature of their experiences. • Young people felt they needed to develop rapport with clinicians before sharing sensitive information. • Clinicians reported a tension between fluid nature of patient experiences and standard format of PROM. • Parents of children with diabetes were concerned that focusing consultations on emotional issues might mean less time was available for discussing diabetes management.
Sharples et al. [27]	Standardised PROMs used within child and adolescent mental health services in one mental health trust in the UK	Semi-structured interviews with nine clinicians in from CAMHS in one mental health trust in London, UK		✓			✓	✓		• Clinicians felt mandatory use of PROMs had a negative impact on clinician therapist relationships by taking time away from sessions, not considering differences in client needs and could alienate patients • Clinicians felt patients liked the measured because they provided a structured framework for discussion
Stasiak et al. (2012)	Range of standardised PROMs in routine use to monitor outcomes in child and adolescent mental health services in New Zealand	Five focus groups with 34 children and 21 family members to explore experiences and views of PROMs use for routine outcomes monitoring		✓			✓	✓		• Young people questioned whether the PROMs captured the dynamic and changing nature of their symptoms. • Young people felt completing a PROM was easier and less embarrassing than talking to a clinician about difficult issues but also felt PROMs should be administered by someone they had developed rapport with. • Parents felt completing a PROM signalled someone was taking their concerns seriously but wanted reassurance these data were going to inform the care of their child.
Cheyne and Kinn [67]	Use of the SEIQoL with 20 clients receiving in drug and alcohol counselling service over six months as part of an RCT	Three focus groups with three counsellors to explore SEIQoL use at 12 weeks, 18 weeks and 24 weeks, completion of a questionnaire and review of patient case notes		✓		✓	✓		✓	• Counsellors perceived that SEIQoL completion enabled service users to reflect on their life and identify issues to tackle in therapy. • Counsellors felt use of the SEIQoL prompted them to listen and reflect more on what the service user was saying and supported the process of relationship building with service users.
Alves et al. [19]	Use of two standardised measures - the Treatment Outcomes Profile (TOP), Clinical Outcome Routine Evaluation-Outcome Measure (CORE-OM) and two individualised measures – Psychological Outcomes Profile (PSYCHLOPS) and the Personal Questionnare(PQ)in drug and alcohol services in Portugal	One focus group with ten service users; eight who had completed the measures on entry to treatment only and two had also completed them at 7 months		✓		✓	✓	✓	✓	• Service users felt use of the PROMs had helped them reflect on their clinical situation • Individualised measures were perceived to provide the freedom to talk about any topic, whether it was related to substances use or not • Standardised PROMs raised awareness about the quantity of alcohol or drugs used
Studies exploring the use of PROMs during initial assessment and follow up in palliative care or oncology settings										
Hagelin et al. (2007)	Nurses use of the EORTC QLQC-30 during assessments within in-patient palliative care in Sweden	Questionnaire with closed and open ended questions to explore 26 nurses’ experiences of using the EORTC QLQC-30			✓	✓		✓		• Nurses perceived the EORTC QLQC-30 captured issues patients may not speak about verbally and provided a useful structure to their discussion with patients • Nurses felt EORTC QLQC-30 was more useful when accompanied by a discussion with the patient and was a complement but not a substitute for other nursing assessments.
Mills et al. (2008;[71])	Completion of a weekly diary for 16 weeks comprising the EORTC-QLQC-30 by patients with inoperable lung cancer in three centres in Northern Ireland. No formal feedback of diary data to clinicians	RCT: diary completion but no feedback vs no completion. Primary outcomes: HRQoL measured by FACT-G. Structured questionnaire to assess use at 8 and 16 weeks. Semi-structured interviews with 7 patients who completed the diary.			✓		✓	✓		• Intervention group had worse total FACT-G scores compared to the control group • 60% patients said it was useful to complete the diary but only 23% of patients reported explicitly sharing the diary with clinicians • Reasons for not sharing included clinicians not asking about it, forgetting to take it to clinic and feeling they could tell clinicians hoe they felt without the diary.
Slater and Freeman [66]	Nurses use of the palliative care outcome scale (POS) in a day hospice in UK	One focus group with nine patients who has been using the POS for at least three months to explore their experiences			✓	✓		✓		• Patients felt completion of the POS helped them to reflect on their feelings • Patients perceived the POS was useful in helping them share their concerns with nurses • Some patients felt some of the questions were too distressing to answer
Slater and Freeman [77]	Nurses use of the palliative care outcome scale (POS) in a day hospice in UK	Focus group with eight nurses to explore their experiences of using the POS			✓	✓		✓		• Nurses perceived that patients may not report issues on the POS but reveal them through verbal interaction. • Nurse felt talking to patients more beneficial than asking them to complete the POS. • Nurses felt completing the POS could be distressing for patients who may not wish to confront their problems directly.
Hughes et al. [78]	Clinicians use of the palliative care outcome scale (POS) in the UK	Qualitative telephone interviews with a purposive sample of 22 people who had experience of using the POS in the routine care of patients;			✓	✓		✓		• Clinicians felt the POS was difficult to use when patients were too ill and perceived some patients found it intrusive or upsetting. • Some clinicians found the questions ‘difficult to ask’ or found the responses ‘difficult to deal with’. • Clinicians altered, reworded or omitted items to adapt the POS for use in local circumstances
Hughes et al. [79]	The use of the POS in non-specialist palliative care settings in the UK	Interviews with 13 members of staff and 3 patients to explore use of POS			✓	✓		✓		• Of 25 sites invited to implement the POS, 15 agreed but four subsequently withdrew. • Anticipated recruiting 240 patients across 11 sites; 21 patients actually recruited. • Participating sites perceived use of POS as a research exercise rather than part of routine practice. • Some nurses reluctant to use the POS as because they were concerned it may raise issues they were ill-equipped to manage. • Nurses expressed concerns that asking patients to complete the POS would ‘tar’ their relationship with the patient
Eischens et al. [86]	Eight nurses randomly assigned to implement either the McGill Quality of Life Questionnaire or the Hospice Quality of Life Index-Revised (HQLI) during patient assessments for one week in a hospice setting in the US. In the subsequent week they utilised the other PROM.	Eight nurses interviewed one week after PROMs administration to explore their experiences of use.			✓	✓		✓		• For the MQOL, nurses felt ‘a good rapport was essential for the patients to answer the questions truthfully’ • For the HQLI, nurses perceived it would be too overwhelming for patients. • All nurses reported the MQOL had enabled them to identify an area of patient care they had overlooked but only one nurses reported the HQLI had provided new information about a patient.
Gamlen and Arber [84]	Specialist nurses use of the Symptoms and Concerns Checklist (SCC) during their first assessments with patients with cancer in a community setting in the UK	Semi structured interviews and non participant observation of six specialist nurses conducting first assessments with patients with cancer in the community using the SCC.			✓	✓		✓		• Nurses placed great importance on hearing the patient’s story in their own words as the words patients used provided insight into how they were coping. • Some nurses felt that the SCC constrained the relationship building process with patients • Nurses delayed completion of the form until they had developed rapport with patients • SCC was useful to validate what nurses had gathered through verbal interaction and prompted them to explore some of the non-physical aspects of the patients’ experiences.
Annells and Koch [68]	Pilot study of the implementation of two PROMs during first assessments and follow up in a district palliative care service in Australia over a 40 week period. 59 patients randomly allocated to be assessed using either the McGill Quality of Life Questionnaire and the Client Generated Index.	Eight nurses made notes following each initial (*n* = 59) and follow up (*n* = 8) assessment and were interviewed. Notes and interviews were qualitatively analysed.			✓	✓	✓	✓	✓	• CGI was recommended as the most appropriate tool for use at first assessments but was not recommended for use at follow up • Completion of the CGI encouraged patients to reflect on their life in relationship to their current situation, gave patients ‘permission to be emotional’ and allowed them to ‘tell their story’. • Nurses perceived completion of the CGI provided new information that they would not have usually uncovered within their assessments.
Kane et al. (2017)	The use of the Integrated Palliative Care Outcomes Scale with 25 patients in a palliative care service for people with Chronic Heart Failure in Ireland	Four nurses worked in the clinic and were interviewed about their experiences of using the measures. Eighteen patients who completed the intervention were interviewed about their experiences of using the PROM			✓	✓		✓		• Nurses felt the IPOS provided a comprehensive review of patients needs, especially psychosocial concerns, and opened up a conversation • Nurses had some concerns about the IPOS opening up conversations on topics they did not know how to deal with • Patients felt IPOS gave them a vocabulary to explain their experiences and gave them permission to raise these with clinicians • Patients felt PROMs completion helped them to reflect on their symptoms and what they could do to manage them
Krawczyk and Sawatzky (2017)	Palliative care team selected PROMs and PREMs to implement in a palliative care service including the Edmonton Symptom Assessment-Revised, the McGill Quality of Life Questionnaire, the Canadian Health Evaluation Project Lite Questionnaire. The PROMs were collected using a tablet with patients over a nine week period	Three focus group with three groups of clinicians, interviews with two nurses two weeks after the start of the project and at completion, interviews with three patients, 50 h of observations of nurses and patient use of PROMs over 9 week period			✓		✓	✓		• Nurses preferred to sit with patients while they completed the PROMs as this enabled them probe patients’ answers and engage in dialogue • Patients felt the PROMs enabled them to articulate their feelings • Nurses felt that using the PROMs as an object of ‘mutual focus’ created a shared space where conversations about patients experiences could occur, which in turn helped nurses to build relationships with patients. • Nurses reported using some of the questions in their routine verbal interactions with patients.
Krawczyk et al. [24]	Palliative care team selected PROMs and PREMs to implement in a palliative care service including the Edmonton Symptom Assessment-Revised, the McGill Quality of Life Questionnaire, the Canadian Health Evaluation Project Lite Questionnaire. The PROMs were collected using a tablet with patients over a nine week period	Five focus groups with clinicians and 24 interviews with clinicians								•
Kettis-Linblad et al. [69]	Non-routine use of the SEIQoL-DW by oncologists caring for patients with gastro-intestinal cancer in two hospitals in Sweden.	Qualitative interviews with eight oncologists and 20 patients to explore their experiences of using the SEIQoL			✓	✓			✓	• PROMs completion provided an opportunity for self-reflection, which increased patients’ self-awareness and helped patients feel that the doctor is willing to listen to them. • Doctors used the SEIQoL to increase their knowledge of the patient and some discussed the findings with patients. • Doctors felt the SEIQoL gave a more complete picture of the patient
Testing theory 2										
Chen et al. (2013)	Systematic review of quantitative studies evaluating the feedback of PROMs data in oncology settings	Developed a theory of change to inform search strategy, outcome indicators and inclusion and exclusion criteria. Carried out a narrative synthesis of included studies			✓	✓	✓	✓		• Included 27 studies; 16 RCTs, 2 before & after, 9 observational studies • 23 studies examined the impact of PROMs feedback on patient provider communication; 21 studies reported some positive effect. • 11 studies examined the impact of PROMs feedback on monitoring treatment response; all 11 studies reported a positive impact • 15 studies examined impact on patient outcomes, 13 reported a positive effect.
Basch et al. (2016[20])	Weekly web based electronic symptom monitoring using National Cancer Institute’s Common Terminology Criteria for Adverse Events in patients receiving chemotherapy for cancer in one centre in the US.	Two arm RCT usual care vs web based PROMs collection & feedback;.			✓		✓	✓		• More patients in intervention arm (21%) experienced clinically significant improvement in HRQoL compared to usual care (11%) • Fewer patients in intervention arm (28%) experienced clinically significant worsening in HRQoL compared to usual care (37%). • Patients in intervention arm had higher median survival (31.2 months) compared to usual care (26 months)
Velikova et al. (2004)	Regular touch screen completion of EORTC QLQC-30 and HADs immediately prior to clinic visits for patients with cancer receiving chemotherapy in one centre in the UK.	Three arm RCT; PROMs completion & feedback vs PROMs completion alone vs no PROMs completion..			✓		✓	✓		• Intervention patients showed greater improvements in HRQoL compared to control arm but not the attention control arm. • Number of symptoms discussed greater in intervention than control arm but no differences in number of non-specific functional issues discussed. • Clinicians explicitly referred to HRQoL data in 66 out of the 103 (64%) intervention encounters. • Improvements in patient well-being associated with explicit use of HRQoL data in the consultation.
Takeuchi et al. [93]	Regular touch screen completion of EORTC QLQC-30 and HADs immediately prior to clinic visits for patients with cancer receiving chemotherapy in one centre in the UK.	Secondary, longitudinal content analysis of tape recorded consultations in control and intervention arms of Velikova et al. (2004) RCT. Data from 198 patients who completed four consecutive consultations with one of 28 oncologists; in total 792 consultations were included in the analysis.			✓		✓	✓		• Difference in the number of symptoms discussed between control and intervention groups largest at first consultation. • No difference in number of functional or psychosocial issues discussed between control and intervention arms • Severity of symptoms predictive of whether discussed but no relationship between severity of functional problems and discussion. • Patients predominantly initiated discussions about symptoms and functional problems.
Detmar et al. [88]	Paper and pencil completion of the EORTC-QLQC-30 immediately prior to clinic visits for patients with cancer receiving chemotherapy in one centre in the Netherlands.	Crossover RCT PROMs completion vs no PROMs completion.			✓		✓	✓		• Statistically significant difference in total number of HRQoL issues discussed in fourth consultation between intervention arm (mean 4.5) and control arms (mean 3.7). • No differences in patient management between control and intervention arms • Two of the eight subscales on the SF-36 showed statistically significantly better function in the intervention arm compared to the control arm.
Berry et al. [92]	Touch screen completion of the Electronic Self Report Assessment-Cancer (ESRA-C) immediately before two clinic visits by patients with cancer receiving chemotherapy in two centres in the US.	RCT comparing PROMs feedback vs usual care. Primary outcome was discussion of symptoms and quality of life issues (SQLI)			✓		✓	✓		• When symptoms were reported at ‘threshold’ (severe) levels, they were more likely to be discussed in the intervention than the control arm. • Some symptoms were commonly discussed in both arms, irrespective of whether they were at threshold.
Detmar et al. [88]	No PROM used	Survey of 273 patients undergoing chemotherapy and 10 oncologists to explore their preferences and self-reported behaviour for raising and discussing issues			✓					• Patients’ and clinicians’ perceptions about who is responsible for initiating discussion about HRQoL issues within the consultation vary depending on the issue. • Patients wanted to discuss and clinicians were willing to initiate discussions about physical symptoms. • Fewer doctors saw it as their primary task to initiate discussion of emotional issues and none reported initiating a discussion about these issues by themselves.
Taylor et al. [94]	RCT comparing no PROMs completion with completion of EORTC QLQC-30 but no feedback to clinicians but data treated as a cohort study	Patient questionnaire to explore their preferences for discussion of issues. Content analysis of consultations between 212 patients who had four complete consultation recordings and 36 doctors to examine actual behaviour.			✓					• Clinicians and patients have different preferences for raising and discussing social and emotional issues. • Both patients and doctors preferences indicated that they saw it as more appropriate to raise emotional rather than social functioning issues with doctors. • In reality, social functioning was more likely to be discussed within the consultation than emotional functioning
Velikova et al. [98]	No PROM used	Eight focus groups with 31 patients and four focus groups with 16 clinicians to explore their views on the use of PROMs in clinical practice.			✓					• Clinicians perceived emotional issues are not routinely discussed in consultations with doctors. • Patients felt it was the doctor’s role to treat the cancer and the nurse’s role to address emotional issues. • Clinicians expressed concerns about PROMs raising topics that they could not treat
Absolom et al. [90]	No PROM used	Interviews with six clinical nurse specialists, eight oncologists, four surgeons and five ward sisters to explore current roles and responsibilities in detection and management of emotional distress (ED)			✓					• Detection and management of emotional distress was more often undertaken by nurses, and was seen as more clearly part of their remit • Oncologists and surgeons prioritised cancer treatment and the management of ED was not considered a key part of their role • Oncologists and surgeons felt able to address emotional distress related to cancer and its treatment but broader emotional problems were seen as the nurse’s role.
Greenhalgh et al. [30]	Regular touch screen completion of EORTC QLQC-30 and HADs immediately prior to clinic visits for patients with cancer receiving chemotherapy in one centre in the UK. Graphical representation of patients’ scores over time provided to oncologist during clinic visit.	Analysis of purposively selected 18 consultations from the intervention arm and four consultations from the attention control arm using conversation analysis to explore how clinicians referred to HRQoL data			✓		✓	✓		• Doctors had to reconcile patients’ verbal reports of symptoms and their PROMs scores. • Patients invited to account and explain their PROMs scores • If high PROMs scores was not a problem for the patient or was not related to cancer, conversation tended to be closed down.
Green et al. [22]	Routine collection of the Edmonton Symptom Assessment System (ESAS) across 14 regional cancer centres in Canada	Survey of 960 clinicians to assess their views of using the ESAS in routine patient care. Study reports findings from 353 nurses who responded			✓	✓	✓	✓		• 59% of nurses agreed ESAS improves the efficiency of their meeting with patients • Free text comments also indicated nurses has concerns that ESAS may length the visit when patients report symptoms not related to cnacer • 84% nurses agreed ESAS was a useful starting point to assess patient symptoms; free text comments emphasised it opened up a conversation about symptoms
Pereira et al. [26]	Routine collection of the Edmonton Symptom Assessment System (ESAS) across 14 regional cancer centres in Canada	Survey of 960 clinicians to assess their views of using the ESAS in routine patient care.			✓	✓	✓	✓		• 67% of physicians agreed or strongly agreed ESAS helps patients report their symptoms compared to 84% of PSO staff • 43% of physicians agreed or strongly agreed that ESAS improves the efficiency of the meeting compared with 60% of nurses • 67% of physicians agreed that ESAS acts as a useful starting point to assess symptoms compared to 95% of PSO staff. • 79% of physicians indicated they always or often looked at patients’ ESAS scores compared with 89% of nurses. • 61% of physicians said they always or often talked about ESAS symptoms to their patients compared to 85% of nurses.

### Theory 1: PROMs support patients to raise issues with clinicians

Across all contexts, we found evidence to support the theory that PROMs completion prompts patients to engage in self-reflection [21, 25, 26, 67–71], enables them to identify what is important to them and develop a deeper understanding of how their condition has affected their life [69–72]. However, this depended on the care context; in palliative care, patients found PROMs completion an emotional experience and the degree to which they engage may depend on their preferred coping strategy; patients who cope by denying their current situation may avoid completing PROMs or not report the true extent of their feelings [68]. Furthermore, frequent PROMs completion for terminally ill patients without formal channels of feedback to clinicians can reduce patients’ HRQoL [73]. In contrast, being asked to complete a PROM when responses are fed back to clinicians can signal to the patient that they feel someone is interested in their feelings [67, 68, 74, 75] and gives them ‘permission’ to share or raise issues with clinicians [25, 69–71].

We then tested whether the structure of the PROM shaped patients’ experiences of completing them and how well patients and clinicians perceived they captured patients’ problems. In primary care and specialist mental health care, some patients felt that standardised PROMs simply did not fully capture the complexity or dynamic nature of their symptoms, particularly for patients with mental health problems [67, 75, 76]. These observations were shared by clinicians [67, 77–79] who expressed concern that the wording of some PROMs upset or alienated patients [29, 80, 81]. Those studies that directly compared individualised and standardised PROMs found that patients felt the former had greater validity and were less distressing [82]; clinicians also preferred individualised measures [70]. However, qualitative studies have noted that cues are co-produced by patients and clinicians during individualised PROMs completion [83] and the process of reducing these cues to a score can result in a loss of meaning [83, 84].

Next, we examined how PROMs structure and care context shaped patients’ and clinicians’ experiences of PROMs as a means for patient to raise issues with clinicians. In primary care, some patients felt that the ‘impersonal’ nature of standardised PROMs was helpful in enabling them to share issues [67]. Similarly, in specialist mental health care, clinicians [29] and service users [21] perceived that patients liked the structured nature of PROMs as it gave a framework for discussion and made talking about problems easier. While patients were generally supportive of the use of standardised PROMs as means of enabling them to share their experiences, clinicians expressed some reservations. In primary care, GPs perceived the use of standardised PROMs for identifying patients with depression as detrimental to clinician-patient communication because they ‘trivialised’ patients’ emotions and resulted in ‘bombarding’ patients with questions in a ‘mechanistic’ way [77, 78, 85]. GPs also found it difficult to incorporate PROMs completion and review into the natural flow of consultations [77, 85]. In specialist mental health care, clinicians expressed concern that asking patient to complete PROMs to comply with the reporting requirements of the Improving Access to Psychological Therapies programme when clients did not wish to complete them was detrimental to the therapeutic alliance [29]. In palliative care, the picture was mixed. In some studies, nurses perceived standardised PROMs constrained relationship building when they were used during first assessments [86] or routine visits [80, 81]. The difficulties clinicians reported echoed the ‘interactional strangeness’ of the standardised survey interview, where standardisation is required to support the psychometric validity of the PROM but at the same time restricts opportunities for sense making [87]. However, other studies found that when standardised PROMs were completed together by the clinician and patient on a tablet, this opened up opportunities for a conversation about the patients answers [26].

We found some evidence to suggest that in palliative care and mental health care, individualised PROMs were perceived as supporting communication by enabling the patient to tell their story in their own words [21, 69, 70]. However, clinicians struggled to use the scores produced to track change over time, as the issues patients nominated changed [70]. These findings mirror studies of individualised PROMs completion outside of clinical settings discussed previously [83, 84]. They provide further lateral support to Theory 1; suggesting that the process of PROMs completion is the stimulus of discussion, rather than the score itself.

Finally, we explored how clinician-patient relationships shaped the ways in which clinicians used PROMs in their interactions with patients. We found that across all care contexts, clinicians and patients felt that having a trusting relationship was necessary to support the sharing of concerns and problems [67, 70, 75–79, 85, 86, 88]. Clinicians placed great emphasis on developing rapport and a trusting relationship with patients through verbal interactions and preferred to let patients ‘tell their story’ in their own words [27, 77, 78, 85, 86]. In secondary mental health care, patients were reluctant to share their feelings through PROMs completion until this relationship had been developed [75, 76]. In palliative care, clinicians used a number of strategies to manage the process of completing a PROM in a way that preserved their relationship with patients. These included completing the PROM alongside patients[26], delaying the use of standardised PROMs to assess patients’ needs during their interactions with patients until they perceived a relationship had been sufficiently built [86], avoiding using them at all [81] or omitting or changing items to avoid upsetting patients [80]. Thus, clinicians adapted their use of PROMs to render them compatible with the ongoing management of patient relationships.

### Theory 2: PROMs raise clinicians’ awareness of patients’ problems

Theory 2 hypothesised that PROM scores alert clinicians to patients’ problems and in turn prompt discussion and subsequent care processes. To test and refine this theory we began by identifying patterns in the impact of PROMs on communication and patient outcomes in oncology within an existing systematic review [8]. First, we explored whether there were any similarities in context between the RCTs that demonstrated a positive impact on patient outcomes and those that revealed no impact. We identified a notable shift in the type of PROM used to provide feedback to patients within RCTs over time; earlier trials evaluated the use of PROMs which measured patients’ functioning, HRQoL and symptoms [89, 90] whereas more recent trials have largely fed back symptom measures [23, 91]. In addition, in earlier trials, PROMs data were fed back to the clinician just before or during the consultation and provided additional information to inform discussion, whereas in later trials, feedback occurred in between clinic visits, enabling more frequent monitoring of patients. Chen et al. [8] found that of the 15 studies that reported any impact of patient outcomes, 13 reported some positive effect, of which nine involved the feedback of symptom measures, rather than HRQoL. Improvements in physical symptoms and chemotherapy side effects were most common. Feedback often occurred between clinic visits and thus acted as a substitute for more frequent follow up. An RCT published subsequent to the systematic review showed a similar pattern, revealing that feedback of patient reported symptoms and side effects resulted in reduced symptom severity, improved health related quality of life (as measured by the EQ-5D), receipt of active chemotherapy for longer, reduced visits to the emergency department and increased survival [22, 23]. Two studies from the systematic review found no impact on outcomes both involved the feedback of HRQoL measures [92, 93]. This tentatively suggests that clinicians may be more likely to respond when feedback provides them with information on symptom severity rather than on HRQoL and when it acts as a substitute, rather than an addition, to clinical encounters.

To further test this emerging explanation, we conducted a more detailed analysis of the impact of PROMs feedback on communication within the studies included in Chen et al. [8] review. Chen et al. [8] found that 21 out of 23 studies reported a ‘positive effect’ on communication. However, who was asked and the ways in which this positive impact was measured varied. The majority of studies relied on retrospective single item questions of satisfaction with communication or on questionnaire surveys and interviews. A small number of trials, which examined the feedback of PROMs during systemic cancer therapy, audio-recorded consultations and subjected them to content analysis [89, 90, 94, 95]. What participants think or recall being discussed or how they experience an interaction can depend on who is asked (the patient or the clinician) and can be different to what the analysis of tape recordings reveal was actually discussed [96–98]. Few of the trials adopted more than one method. In our synthesis, we focused on those studies that had conducted a detailed analysis of interactions within oncology consultations. This enabled us to explore subtle but important variations in what was discussed, by whom and when.

Takeuchi et al. [95] conducted a detailed analysis of consultations recorded within Velikova et al’s [89] trial involving feedback of the cancer specific HRQoL measure (EORTC QLQC-30) and the Hospital Anxiety and Depression Scale. They found that the difference in the number of symptoms discussed between control (no PROM) and intervention groups was largest during the first consultation, suggesting PROMs are most likely to provide ‘new’ information to the clinician at this point. They found no differences in the number of functional impairments discussed. While the severity of symptoms was predictive of whether they were discussed, there was no relationship between the severity of functional impairments and the likelihood of them being discussed. They also observed that discussion of symptoms and functions were predominantly initiated by patients (with the exception of dyspneoa and bowel habits) and that PROM feedback did not prompt oncologists to increase their enquiries about patients’ problems. Similarly, Berry et al. [94] found that whether symptoms were discussed depended on their severity. However, clinicians continued to focus on common side effects of chemotherapy, irrespective of whether or not these were reported as a severe problem by patients.

These findings provide some support to theory 1, that PROMs may support clinician-patient communication through giving patients ‘permission’ to raise issues with clinicians. They also suggest that although PROMs may not lead to clinicians initiating a discussion about either patients’ symptoms or functional status, they enable clinicians to identify symptoms that are particularly severe for patients. They further indicate that patients’ functional status is less likely to be explicitly discussed as a result of PROMs feedback than symptoms or biomedical issues and that the overall focus of the consultation remains on the management of chemotherapy side effects and reviewing treatment effectiveness. The next phase of our synthesis examined possible explanations for these findings by reviewing qualitative studies and surveys that explored patients and clinicians’ experiences and views of using PROMs in cancer care.

One explanation is that clinicians or patients (or both) do not feel that consultations during chemotherapy are an appropriate context for discussion of emotional, social or non-biomedical issues [95]. Detmar et al. [99] found that patients and doctors saw physical symptoms as clearly within the doctor’s remit and would be willing to discuss them in the consultation while none of the doctors would be willing to initiate discussion about emotional issues on their own. Taylor et al. [96] found that although many patients and doctors felt it appropriate to raise discussion about emotional issues, in reality, emotional issues were only mentioned in 27% of consultations and led to a discussion in less than half of these instances. While fewer patients and clinicians felt it was appropriate to raise concerns about social functioning in the consultation, these issues were actually raised more frequently (46% of the time) than emotional problems [96]. The authors hypothesise that this is because problems with social functioning are more likely to be caused by the physical impact of cancer, which oncologists see as the within their remit and the purpose of the consultation. Surveys have also found that clinicians were concerned patients may raise issues not related to cancer [24].

Another explanation of these findings is that doctors do not see the explicit discussion of functional or emotional issues as falling within their remit [62]. Surveys of cancer care professionals have shown that a higher percentage of nurses expressed positive attitudes to the value of PROMs in supporting patient care compared to physicians [28]. Qualitative studies have found that patients see the doctor’s remit as focusing on biomedical issues and are unsure whether it is the doctor’s role to address emotional or functional issues [100, 101]. Doctors also acknowledged that emotional issues are not routinely discussed and would not enquire about them unless patients volunteered information. Doctors, especially surgeons, felt their remit was treating the patient’s cancer and although they felt able to deal with emotional issues related to clinical problems, they felt that it was the nurses’ role to address wider emotional issues, a view shared by nurses [101]. Similarly, Greenhalgh et al. [32] observed that PROM scores do not distinguish between ‘problems related to cancer’ and ‘other problems’. As a result, oncologists had to reconcile between PROMs scores and patients’ verbal reports by inviting patients to account for high PROMs scores. Thus, to make sense of PROMs scores, clinicians needed to explore how and why patients had arrived at their answers. While these strategies opened up a discussion between the patient and the doctor, these discussions tended to be closed down when patients’ accounts revealed that the issue was not problematic for them or was not related to cancer.

## Discussion

In this section we discuss and explain the main findings of our review in the context of broader debates about meaning in survey completion and clinician-patient communication. We note the limitations of our review and finally consider the implications of our findings for the use of PROMs in clinical practice.

### Main findings

With regards to the theory that PROMs completion supports patients to raise issues with clinicians (Theory 1), we found that, for both standardised and individualised PROMs, the process of PROMs completion prompts patients to reflect on their health and in doing so, patients develop a deeper understanding of how their condition affects them. We also found that PROMS completion can enable patients to raise issues with clinicians by providing a framework for discussion and giving them ‘permission’ to raise issues, as it can signal that the clinician is interested in their views. This suggests that the process of PROMs completion is not simply a task of information retrieval, nor is it a neutral, inert activity of obtaining structured, standardised information from patients. Rather, the ways in which patients interpret questions and construct their answers is shaped by social and cultural factors and can affect the ways in which patients understand, frame or think about their condition [49, 87]. Drawing on the work of Gadamer [122], McClimans [123] offers a theoretical account of the PROMs completion process that can explain our findings. She argues that PROMs ask ‘genuine questions’, that is, questions which open up inquiry into the subject matter at hand but also the meaning of that subject matter. In order to answer a PROM item, respondents must infer both the subject matter of the question and the meaning of the subject matter implied by the question. McClimans [123] and others [102] observe that respondents bring their own understandings of that construct to bear on the question and attempt to understand PROMs items by relating these items to their own lives. In doing so, they may find that their understanding of that subject matter, that is, how their condition is affecting their symptoms, functioning and health related quality of life, is transformed. Thus, it is through these processes that PROMs provide an opportunity for respondents to reflect on their health and come to a deeper understanding of how their own condition affects them or what is most important to them. Furthermore, when patients are asked to complete a PROM, they often assume that this is because the clinician is interested in the findings [103]; this may signal to the patient that items contained in the PROM are appropriate topics for discussion, thus giving patients ‘permission’ to raise them.

In contrast, clinicians across a range of clinical settings found using a standardised PROM during initial assessments could constrain, rather than support communication and interfered with the process of managing relationships with patients, while individualised PROMs supported this dialogue. The ways in which PROMs data are socially produced can also explain how and why the structure of the PROM shapes clinicians’ experiences of using PROMs in clinical practice. As Mallinson [87] noted, when a PROM is completed within an interview, an additional layer of social interaction is brought into play. Standardised PROM completion is unlike the usual flow of conversation and is different to the interaction which occurs within consultations [104]. The direction of questioning is one way and the wording of the questions should not be altered, otherwise the validity of the PROM, as underpinned by psychometric testing, is threatened. Thus, as Mallinson [87] observes, the standardised survey interview creates an ‘interactional strangeness’ where ‘most of the mechanisms to check meaning are supressed’. Other studies have also found that standardised checklists and frameworks can narrow discussion and disrupt the process of managing and building relationships with patients [33, 105, 106]. In contrast, Krawczyk et al. [26] showed that when standardised PROMs were used in a ‘relational’ way, that is, nurses sat with patients as they completed the PROM and probed patients answers as they were produced, a dialogue with the patient was opened up. Similarly, individualised PROMs, appeared to mimic the more open structure clinicians used in their interactions with patients and allowed patients to ‘tell their story’ in their own words and provided opportunities to check meanings. Thus, it is the interaction between the material properties of the PROM and the existing social relations that shape how the support or detract from clinician patient relationships [26].

We tested theory 2 by focusing on oncology, where, in the majority of studies, patients completed a PROM prior to the consultation. PROMs act like a test result that prompts clinicians to discuss problems with patients and offer support for symptom management when the PROM functioned as a substitute, rather than an addition to the clinical encounter and when the PROM focused on symptoms and side effects rather than HRQoL. Following PROMs feedback, consultations with doctors largely focused on symptoms and side effects, rather than on patient functioning. Patients did not always feel it was appropriate to discuss functional and HRQL aspects with doctors and doctors did not perceive this was within their remit. In contrast, nurses felt discussion of such issues fell to them. These findings reflect the wider literature on communication in oncology [107–110] and have often been interpreted as doctors not being ‘patient centred’ or holding a lack of concern for patients’ emotional well-being [31]. However, recent studies have shown that patients can feel emotionally supported without explicit emotional talk within oncology consultations because they view doctors as experts who have the knowledge and authority to treat them [98, 111, 112]. Salmon and Young [31] argue that rather than seeing biomedical talk as an attempt to avoid engaging emotionally with patients, it represents doctors meeting their responsibility to provide emotional support to patients with cancer by treating the disease.

### Limitations

Our findings for theory 2 may only be generalizable to oncology settings as we tested this theory using only empirical evidence from oncology. To test this theory further, it would be valuable to contrast oncology with the ways in which clinicians respond to PROMs scores in (for example) psychotherapy and specialist mental health care, as explicit emotional discussion is a central feature of therapy. We were not able to do this in our current review due to time and resource constraints. Similarly, we did not review the emerging literature about the use of PROMs to support patient care in orthopaedics [113, 114]. We also recognise that we did not test and refine all of the programme theories underlying how PROMs are thought to support the care of individual patients. For example, we did not explore whether and how PROMs enables shared decision-making or supports patient activation in the self-management of long term conditions [46]. Although we identified some key contextual conditions that shape how PROMs are used, we did not consider how the use of PROMs might be shaped by race, gender and age. We cannot rule out the possibility that an important study was missed from our review. However, the aim of this review was not to conduct an exhaustive search for all studies, but rather to sample those studies that were most relevant to testing and refining our two selected theories. Nonetheless, we specifically included on a wide range of recent and updated systematic reviews of both RCTs and qualitative studies [8, 15, 115]. Accepting these limitations, the review still has important implications for the use of PROMs in clinical practice and future research.

### Conclusions and implications for research and practice

Studies evaluating the impact of PROMs feedback to clinicians on patient-physician communication have rarely considered how the process of PROMs completion impacts upon patients. Our review highlights that this is an important consideration. Exploring how and why patients answer PROMs in the ways that they do, in addition to understanding how clinicians and patients interpret the score itself, can expand our knowledge of how patients understand their condition and its impact [30].

Studies exploring the impact of PROMs on clinician patient communication and care processes have also largely focused on the ways in which PROMs impact on the information sharing and decision-making functions of the consultation, rather than on the impact of PROMs on the relationship building function. Our review indicates that the process of relationship building with patients is affected by and shapes the ways in which clinicians use PROMs in clinical practice. Krawczyk et al. [26] argue that we need to consider the interconnection between social relations and the materiality of PROMs to understand how PROMs support collaboration between patients and clinicians. As Brewster et al. [106] note, clinicians experience measurement tools as being socially situated; their use is entangled with the ongoing work of managing patient relationships. This has an important, but often overlooked, impact on how such measurement tools are used in clinical practice. Those implementing PROMs in clinical practice to support patient management need to consider how PROMs can be introduced in a way that supports, rather than detracts from the clinician-patient relationship. This will entail giving clinicians considerable freedom in how and when they use PROMs and allowing adaption of PROM items to local and disease trajectory specific circumstances.

Our review also suggests that PROMs can enable better identification of and greater discussion of problematic symptoms and side effects during chemotherapy but do not necessarily shift the focus of the consultation onto emotional and functional aspects. To achieve this shift would require a change in clinicians’, specifically doctors’, perceptions of their remit. Professional groups’ perceptions of their remit are socialised through many years of education and training and are mutually reinforced through the division of labour in the everyday practices of different clinical groups [116]. PROMs feedback alone is not sufficient to change these practices, which would require concomitant changes to organisation-wide structures that both produce and reinforce these professional boundaries. While training clinicians in the use and interpretation of PROMs is helpful [117], this does not address the structural constraints which may limit these discussions.

Furthermore, our review suggest that patients may not always want to discuss these issues with doctors; recent studies also indicate that discussion and management of biomedical issues can provide emotional support to patients [111]. Many current measures of patient centred communication assume that the discussion of functional impairments and emotional concerns is an important component of patient centred communication [118]. However, recent reviews [58] indicate that we need to rethink what constitutes patient centred communication and have argued that emotional and instrumental care are inseparable. In an oncology context, for example, patients value clinicians who can treat their cancer [112]. Our review suggests that PROMs feedback can support instrumental and emotional care through enabling clinicians to identify problematic symptoms and through supporting patients to raise concerns about these symptoms. Thus, future research on how PROMs feedback supports clinician patient communication and care processes should explore not what is talked about but *how* it is talked about and how patients experience this care using multiple methods [112].

## Additional files

Additional file 1: Search strategies. (DOCX 18 kb)

Additional file 2: Inclusion and exclusion criteria. (DOCX 12 kb)

## Abbreviations

EORTC QLQC-30: European Organisation for Research and Treatment of Cancer Quality of Life Questionnaire Core − 30
HRQoL: Health Related Quality of Life
PROM: patient reported outcome measure
SEiQoL: Schedule for the Evaluation of Individual Quality of Life
