# Supplementary material for: How do patient reported outcome measures (PROMs) support clinician-patient communication and patient care? A realist synthesis
Source: J Patient Rep Outcomes. 2018 Sep 15;2:42. doi: 10.1186/s41687-018-0061-6 (PMC6153194; doi:10.1186/s41687-018-0061-6)
Supplement: Supplementary file 1 — Search strategies. (DOCX 18 kb) [file 41687_2018_61_MOESM1_ESM.docx]

**Appendix 1: Search strategies**

These search strategies were used to identify theory papers.

- 1. PROMs Feedback Reviews

Ovid MEDLINE(R) <1946 to April Week 3 2014>

Search Strategy:

--------------------------------------------------------------------------------

1 ("patient report*" adj3 (outcome* or data)).tw. (3342)

2 proms.tw. (151)

3 ("public* report*" adj3 (outcome* or data or information or care)).tw. (295)

4 exp *Health Status Indicators/ and (*patient satisfaction/ or *patient preference/ or *Patient-Centered Care/) (189)

5 *"Outcome Assessment (Health Care)"/ and (*patient satisfaction/ or *patient preference/ or *Patient-Centered Care/ or *Professional-Patient Relations/) (791)

6 *Self Report/ and *Quality of Life/ (101)

7 *"Quality of Life"/ and Health Status/ and patient satisfaction/ and ("Outcome Assessment (Health Care)"/ or Questionnaires/) (212)

8 ("public report*" adj3 hospital*).tw. (78)

9 (star adj2 rating*).ti. (22)

10 or/1-9 [PROMs] (4813)

11 exp Decision Making/ (122197)

12 quality indicators, health care/ (9977)

13 exp treatment outcome/ (640336)

14 exp Feedback/ (42542)

15 ((PROM or PROMs or PRO) adj3 acceptab*).tw. (16)

16 (improve* or feedback).ti. (141142)

17 or/11-16 [Feedback theories] (925409)

18 10 and 17 [Proms Feedback] (1865)

19 limit 18 to "reviews (maximizes specificity)" (143)

20 (Review* adj5 outcome*).ab. (13974)

21 (Review* adj7 outcome*).ti. (2133)

22 (Review* adj2 (PRO or PROM or PROMs)).tw. (117)

23 (review* adj3 data).tw. (31333)

24 or/20-23 (46469)

25 18 and 24 (81)

26 19 or 25 (190)

- 1. PROMs Feedback Opinions and Commentaries

Ovid MEDLINE(R) <1946 to April Week 3 2014>

Search Strategy:

--------------------------------------------------------------------------------

1 Comment/ (535128)

2 Letter/ (808856)

3 Editorial/ (336724)

4 news/ or newspaper article/ (169377)

5 "Comment on".ti. (12027)

6 (letter* adj3 editor*).ti. (3046)

7 opinion*.ti. (9839)

8 (view or views).ti. (37996)

9 comment.cm. (1007256)

10 or/1-9 [Commentary and Opinion Pieces] (1886406)

11 ("patient report*" adj3 (outcome* or data)).tw. (3342)

12 proms.tw. (151)

13 ("public* report*" adj3 (outcome* or data or information or care)).tw. (295)

14 *"Outcome Assessment (Health Care)"/ and (*patient satisfaction/ or *patient preference/ or *Patient-Centered Care/ or *Professional-Patient Relations/) (791)

15 ("public report*" adj3 hospital*).tw. (78)

16 (star adj2 rating*).ti. (22)

17 or/11-16 [PROMs] (4403)

18 Patient Participation/ (17911)

19 quality indicators, health care/ (9977)

20 exp treatment outcome/ (640336)

21 (improve* or impact* or transform* or feedback).ti. (321717)

22 exp Feedback/ (42542)

23 *Attitude of health personnel/ (45015)

24 Quality of healthcare/ (56546)

25 or/18-24 [Feedback theories] (1084628)

26 10 and 17 and 25 [Proms Feedback Commentaries] (286)

**Cochrane Database of Systematic Reviews : Issue 5 of 12, May 2014 (Wiley)**

Search Name: PROMS Theories reviews 02-05-2014

Date Run: 02/05/14 08:58:45.325

ID Search Hits

#1 "patient report*" near/3 (outcome* or data):ti 335

#2 proms:ti 33

#3 "public* report*" near/3 (outcome* or data or information or care):ti 2

#4 "public report*" near/3 hospital*:ti 0

#5 (star near/2 rating*):ti 1

#6 #1 or #2 or #3 or #4 or #5 369

#7 (decision* near/3 (shared or patient*)):ti,ab 956

#8 feedback or impact* or transform*:ti 53752

#9 improve* near/3 (care or satisfaction or choice or healthcare):ti,ab 2644

#10 enhance* near/3 (care or satisfaction or choice or healthcare):ti,ab 462

#11 improve* or effective* or efficac*:ti 288493

#12 ((PROM or PROMs or PRO) near/3 acceptab*):ti,ab 2

#13 #7 or #8 or #9 or #10 or #11 or #12 312346

#14 #6 and #13 225

**Cochrane Methodology Register : Issue 3 of 4, July 2012 2014 (Wiley)**

Same strategy as Cochrane Database of Systematic Reviews

**Database of Abstracts of Reviews of Effect : Issue 4 of 4, October 2014 2014 (The Cochrane Library, Wiley)**

Same strategy as Cochrane Database of Systematic Reviews

**NHS Economic Evaluation Database : Issue 4 of 4, October 2014 (The Cochrane Library, Wiley)**

Same strategy as Cochrane Database of Systematic Reviews

**Embase Classic+Embase <1947 to 2014 April 30> - Commentaries Search**

1 letter/ (815613)

2 editorial/ (482060)

3 note/ (550801)

4 "Comment on".ti. (14947)

5 (letter* adj3 editor*).ti. (18574)

6 opinion*.ti. (14096)

7 (view or views).ti. (54267)

8 or/1-7 (1912221)

9 ("patient report*" adj3 (outcome* or data)).tw. (6795)

10 proms.tw. (306)

11 ("public* report*" adj3 (outcome* or data or information or care)).tw. (417)

12 ("public report*" adj3 hospital*).tw. (116)

13 (star adj2 rating*).ti. (26)

14 *public reporting/ (17)

15 (routine outcome adj1 (measure* or assessment* or monitoring)).ti. (98)

16 *patient reported outcome/ (88)

17 *patient reported outcomes measurement information system/ (29)

18 *outcome assessment/ or *symptom assessment/ (10704)

19 patient satisfaction/ or patient preference/ or patient decision making/ or doctor patient relation/ (167151)

20 18 and 19 (821)

21 9 or 10 or 11 or 12 or 13 or 14 or 15 or 16 or 17 or 20 [PROMs] (8209)

22 exp health care quality/ (1943631)

23 feedback.ti. (16514)

24 feedback system/ or negative feedback/ or positive feedback/ (71668)

25 *performance/ or *job performance/ (6874)

26 exp health personnel attitude/ (129925)

27 exp decision making/ (214272)

28 or/22-27 [Feedback outcomes] (2164599)

29 8 and 21 and 28 (258)

**Embase Classic+Embase <1947 to 2014 April 30> - Reviews Search**

1 ("patient report*" adj3 (outcome* or data)).tw. (6795)

2 proms.tw. (306)

3 ("public* report*" adj3 (outcome* or data or information or care)).tw. (417)

4 ("public report*" adj3 hospital*).tw. (116)

5 (star adj2 rating*).ti. (26)

6 *public reporting/ (17)

7 (routine outcome adj1 (measure* or assessment* or monitoring)).ti. (98)

8 *patient reported outcome/ (88)

9 *patient reported outcomes measurement information system/ (29)

10 *outcome assessment/ or *symptom assessment/ (10704)

11 patient satisfaction/ or patient preference/ or patient decision making/ or doctor patient relation/ (167151)

12 10 and 11 (821)

13 1 or 2 or 3 or 4 or 5 or 6 or 7 or 8 or 9 or 12 [PROMs] (8209)

14 exp *health care quality/ (318360)

15 feedback.ti. (16514)

16 feedback system/ or negative feedback/ or positive feedback/ (71668)

17 ((PROM or PROMs or PRO) adj3 acceptab*).tw. (24)

18 *performance/ or *job performance/ (6874)

19 exp *health personnel attitude/ (58426)

20 exp *decision making/ (48342)

21 or/14-20 [Feedback outcomes] (443461)

22 13 and 21 (2130)

23 limit 22 to "reviews (maximizes specificity)" (95)

24 (Review* adj2 (PRO or PROM or PROMs)).tw. (168)

25 (Review* adj7 outcome*).ti. (3041)

26 (Review* adj5 outcome*).ab. (23707)

27 (review* adj3 data).tw. (46731)

28 or/24-27 (71642)

29 22 and 28 (114)

30 23 or 29 (171)

**HMIC Health Management Information Consortium <1983 - present> - Commentaries Search**

1 ("patient report*" adj3 (outcome* or data)).tw. (129)

2 proms.tw. (54)

3 ("public* report*" adj3 (outcome* or data or information or care)).tw. (37)

4 ("public report*" adj3 hospital*).tw. (10)

5 (star adj2 rating*).ti. (46)

6 (routine outcome adj1 (measure* or assessment* or monitoring)).ti. (8)

7 or/1-6 [PROMs] (231)

8 (improve* or feedback).ti. (4538)

9 ((PROM or PROMs or PRO or patient*) adj3 acceptab*).tw. (254)

10 decision making/ (4856)

11 "quality of patient care"/ or "quality of nursing care"/ (9378)

12 feedback/ (161)

13 consumer feedback/ (136)

14 exp patient outcome/ (5115)

15 exp performance/ (14081)

16 exp attitudes/ (18436)

17 or/8-16 (51102)

18 7 and 17 (137)

19 opinion*.ti. (481)

20 (view or views).ti. (3282)

21 editorial.mp. [mp=title, other title, abstract, heading words] (573)

22 note.mp. [mp=title, other title, abstract, heading words] (2001)

23 comment.mp. [mp=title, other title, abstract, heading words] (1020)

24 letter.mp. [mp=title, other title, abstract, heading words] (2607)

25 or/19-24 (9791)

26 18 and 25 (9)

**HMIC Health Management Information Consortium <1983 - present>- Reviews Search**

1 ("patient report*" adj3 (outcome* or data)).tw. (129)

2 proms.tw. (54)

3 ("public* report*" adj3 (outcome* or data or information or care)).tw. (37)

4 ("public report*" adj3 hospital*).tw. (10)

5 (star adj2 rating*).ti. (46)

6 (routine outcome adj1 (measure* or assessment* or monitoring)).ti. (8)

7 or/1-6 [PROMs] (231)

8 (improve* or feedback).ti. (4538)

9 ((PROM or PROMs or PRO or patient*) adj3 acceptab*).tw. (254)

10 decision making/ (4856)

11 "quality of patient care"/ or "quality of nursing care"/ (9378)

12 feedback/ (161)

13 consumer feedback/ (136)

14 exp patient outcome/ (5115)

15 exp performance/ (14081)

16 exp attitudes/ (18436)

17 or/8-16 (51102)

18 7 and 17 (137)

19 review*.mp. (35585)

20 18 and 19 (17)

**Ovid MEDLINE(R) <1946 to April Week 3 2014> - Commentaries Search**

1 Comment/ (535128)

2 Letter/ (808856)

3 Editorial/ (336724)

4 news/ or newspaper article/ (169377)

5 "Comment on".ti. (12027)

6 (letter* adj3 editor*).ti. (3046)

7 opinion*.ti. (9839)

8 (view or views).ti. (37996)

9 comment.cm. (1007256)

10 or/1-9 [Commentary and Opinion Pieces] (1886406)

11 ("patient report*" adj3 (outcome* or data)).tw. (3342)

12 proms.tw. (151)

13 ("public* report*" adj3 (outcome* or data or information or care)).tw. (295)

14 *"Outcome Assessment (Health Care)"/ and (*patient satisfaction/ or *patient preference/ or *Patient-Centered Care/ or *Professional-Patient Relations/) (791)

15 ("public report*" adj3 hospital*).tw. (78)

16 (star adj2 rating*).ti. (22)

17 or/11-16 [PROMS] (4403)

18 Patient Participation/ (17911)

19 quality indicators, health care/ (9977)

20 exp treatment outcome/ (640336)

21 (improve* or impact* or transform* or feedback).ti. (321717)

22 exp Feedback/ (42542)

23 *Attitude of health personnel/ (45015)

24 Quality of healthcare/ (56546)

25 or/18-24 [Feedback theories] (1084628)

26 10 and 17 and 25 [Proms Feedback Commentaries] (286)

**Ovid MEDLINE(R) <1946 to April Week 3 2014>- Reviews Search**

1 ("patient report*" adj3 (outcome* or data)).tw. (3342)

2 proms.tw. (151)

3 ("public* report*" adj3 (outcome* or data or information or care)).tw. (295)

4 exp *Health Status Indicators/ and (*patient satisfaction/ or *patient preference/ or *Patient-Centered Care/) (189)

5 *"Outcome Assessment (Health Care)"/ and (*patient satisfaction/ or *patient preference/ or *Patient-Centered Care/ or *Professional-Patient Relations/) (791)

6 *Self Report/ and *Quality of Life/ (101)

7 *"Quality of Life"/ and Health Status/ and patient satisfaction/ and ("Outcome Assessment (Health Care)"/ or Questionnaires/) (212)

8 ("public report*" adj3 hospital*).tw. (78)

9 (star adj2 rating*).ti. (22)

10 or/1-9 [PROMS] (4813)

11 exp Decision Making/ (122197)

12 quality indicators, health care/ (9977)

13 exp treatment outcome/ (640336)

14 exp Feedback/ (42542)

15 ((PROM or PROMs or PRO) adj3 acceptab*).tw. (16)

16 (improve* or feedback).ti. (141142)

17 or/11-16 [Feedback theories] (925409)

18 10 and 17 [Proms Feedback] (1865)

19 limit 18 to "reviews (maximizes specificity)" (143)

20 (Review* adj5 outcome*).ab. (13974)

21 (Review* adj7 outcome*).ti. (2133)

22 (Review* adj2 (PRO or PROM or PROMs)).tw. (117)

23 (review* adj3 data).tw. (31333)

24 or/20-23 (46469)

25 18 and 24 (81)

26 19 or 25 (190)

**Ovid MEDLINE(R) In-Process & Other Non-Indexed Citations <April 29, 2014> - Commentaries Search**

1 ("patient report*" adj3 (outcome* or data)).tw. (706)

2 proms.tw. (45)

3 ("public* report*" adj3 (outcome* or data or information or care)).tw. (30)

4 ("public report*" adj3 hospital*).tw. (6)

5 (star adj2 rating*).ti. (0)

6 or/1-5 [PROMS] (748)

7 (decision* adj3 (shared or patient*)).tw. (1265)

8 (feedback or impact* or transform*).ti. (23084)

9 (improve* adj3 (care or satisfaction or choice or healthcare)).tw. (3295)

10 (enhance* adj3 (care or satisfaction or choice or healthcare)).tw. (453)

11 exp treatment outcome/ (7)

12 (improve* or effective* or efficac*).ti. (31885)

13 (choice* adj3 (shared or patient*)).tw. (814)

14 ((PROM or PROMs or PRO) adj3 acceptab*).tw. (0)

15 or/7-14 [Outcomes of Feedback] (59317)

16 Comment/ (45363)

17 Letter/ (27707)

18 Editorial/ (16202)

19 news/ or newspaper article/ (8407)

20 "Comment on".ti. (2782)

21 (letter* adj3 editor*).ti. (2925)

22 opinion*.ti. (748)

23 (view or views).ti. (3055)

24 comment.cm. (57845)

25 or/16-24 [Commentary and Opinion Pieces] (101533)

26 6 and 15 and 25 (10)

**Ovid MEDLINE(R) In-Process & Other Non-Indexed Citations <April 29, 2014>- Reviews Search**

1 ("patient report*" adj3 (outcome* or data)).tw. (706)

2 proms.tw. (45)

3 ("public* report*" adj3 (outcome* or data or information or care)).tw. (30)

4 ("public report*" adj3 hospital*).tw. (6)

5 (star adj2 rating*).ti. (0)

6 or/1-5 [PROMS] (748)

7 (decision* adj3 (shared or patient*)).tw. (1265)

8 (feedback or impact* or transform*).ti. (23084)

9 (improve* adj3 (care or satisfaction or choice or healthcare)).tw. (3295)

10 (enhance* adj3 (care or satisfaction or choice or healthcare)).tw. (453)

11 exp treatment outcome/ (7)

12 (improve* or effective* or efficac*).ti. (31885)

13 (choice* adj3 (shared or patient*)).tw. (814)

14 ((PROM or PROMs or PRO) adj3 acceptab*).tw. (0)

15 or/7-14 [Outcomes of Feedback] (59317)

16 (Review* adj5 outcome*).ab. (2125)

17 (Review* adj7 outcome*).ti. (322)

18 (Review* adj2 (PRO or PROM or PROMs)).tw. (8)

19 (review* adj3 data).tw. (2889)

20 or/16-19 (5200)

21 6 and 15 and 20 (11
