# Supplementary material for: How do patient reported outcome measures (PROMs) support clinician-patient communication and patient care? A realist synthesis
Source: J Patient Rep Outcomes. 2018 Sep 15;2:42. doi: 10.1186/s41687-018-0061-6 (PMC6153194; doi:10.1186/s41687-018-0061-6)
Supplement: Supplementary file 2 — Inclusion and exclusion criteria. (DOCX 12 kb) [file 41687_2018_61_MOESM2_ESM.docx]

Appendix: Inclusion and exclusion criteria

**Inclusion**

The study contributes to explaining:

- How PROMs may support patients to raise issues with clinicians
- How PROMs may support the relationship building process between patient and clinician
- How PROMs may constrain the relationship building process
- How PROMs may support discussion within the consultation

Plus studies exploring:

- Clinicians’ and patients’ experiences of using PROMs feedback in oncology, palliative care, primary and secondary mental health settings
- Patients’ experiences of completing standardised and individualised PROMs

**Exclusion criteria**

- Study does not add anything or contribute to theory testing and refinement
- Study explores the psychometric properties of PROMs
- The paper reports findings in which a PROM is used as a research tool (e.g. an evaluation of an intervention, a study exploring the HRQoL of specific populations)
- The paper is focused on evaluating the psychometric properties of a PROM
- The paper reviews the psychometric properties of a PROM or collection of PROMs
